# From trial and error to Equator Network

**Published:** 2012-09-30

**Authors:** Mauricio Palacios Gómez

**Affiliations:** Editor Asociado, Revista Colombia Médica - Facultad de Salud, Universidad del Valle

**Keywords:** EQUATOR Network, authorship, CONSORT, reporting guidelines, research methodology

The dissemination of science is grounded on the reliability and value of the research literature that, through an editorial process, expresses the necessary detail to meet the reproducibility and refutably demands that underlie the scientific method. Three roles are defined in this editorial process that the researcher may assume at differing times or simultaneously, from the beginning of his career to the end of professional life: those of author, reviewer and editor.

Authorship is scientific initiation, and despite identifying ourselves with the scientific method in the development of the research, writing and publication of a manuscript are closer to the methodologies of trial and error during the editorial process. Writing courses on scientific articles favor the structuring of information; however, they cannot cover the individuality of methodological design, and depending on the demands and help provided through editorial work, the published article may have a variable quality that does not reflect the methodological rigor and results of the research that was carried out. 

Sooner or later, publishing an article means receiving a call from the journals to be a reviewer of another manuscript in which one might be considered an expert, and for this new role there is no schooling. Review guidelines are sent along with the document to be evaluated and these are used indiscriminately for a laboratory experiment, a clinical trial or a qualitative analysis, among others. Experience counts and an academic background may lead a reviewer to evaluate using more formative recommendations, while a more exacting researcher will highlight the weaknesses and points where the writing needs improvement. The final result of a peer review evaluation can generate conflicting recommendations from two expert arbiters with adequate argumentation. Again, trial and error will lead to improvements in the qualities of the reviewer, considering that this learning process has a rhythm of its own for each researcher.

The editor assumes the two previous roles on numerous occasions until attaining a criterion (trial and error learning) that permits guidance to the author for the best possible outcome while considering the recommendations of the reviewers. From this standpoint the variability and vulnerability of the entire editorial process are very obvious and a search for guidelines that seek to standardize the information published, based on quality criteria becomes mandatory.

The CONSORT Statement^1^ was the first universally accepted writing guide for controlled clinical studies. Later on, guidelines for studies concerning meta-analysis (PRISMA, previously QUORUM)^2^, epidemiology (MOOSE)^3^, observational (STROBE)^4^, diagnostic accuracy (STARD)^5^ and qualitative (COREQ)^6^ were published. Guidelines have been derived from each of them for specific studies (non-inferiority, bioequivalence, etc.); but their increasing numbers have mandated the grouping of them into a single reference site for authors, reviewers and editors: The Equator Network.

The Equator Network was created in 2006 as a British governmental initiative to group communication guidelines for research studies. It thrived by engaging developers of guidelines, editors of scientific journals, research funding organisms, authors, reviewers and other contributors around the world, providing key tools that improve the quality of health research reporting. Towards the future, it is emerging as an observatory of progress in the quality of published scientific information while maintaining its academic base^7^.

Taking into account that the Equator Network relies, so far, on 95 updated reporting guidelines. Colombia Médica invites all its collaborators to consult this website (www.equator-network.org) and to take into consideration these guidelines when preparing, reviewing and editing manuscripts, with the assumption that they serve only as a guideline where not all studies can meet all the defined requirements. Similarly, all training programs for health researchers should adopt the guidelines of this network for master´s theses and degree-required research reports, so that universal, updated quality criteria for disseminating the effort implied by each investigation are maintained. Ultimately this initiative will reduce trial and error learning in editorial processes.
